# Matrix metalloproteinase 9 is involved in airway inflammation in cough variant asthma

**DOI:** 10.3892/etm.2014.1903

**Published:** 2014-08-13

**Authors:** HUI-PING MA, WEI LI, XIAO-MIN LIU

**Affiliations:** 1 Department of Respiratory Medicine, Jining No. 1 People’s Hospital, Jining, Shandong 272111, P.R. China; 2 Department of Otorhinolaryngology, Jining No. 1 People’s Hospital, Jining, Shandong 272111, P.R. China; 3 Department of Respiratory Medicine, The First Affiliated Hospital of Harbin Medical University, Harbin, Heilongjiang 150001, P.R. China

**Keywords:** cough variant asthma, matrix metalloproteinase 9, inflammation

## Abstract

Previous studies have revealed the role of matrix metalloproteinase 9 (MMP9) in asthma and chronic obstructive pulmonary disease (COPD). However, its role in airway inflammation in cough variant asthma (CVA) remains unknown. In the present study, variations in the levels of MMP9 and interleukin (IL)-5 in the induced sputum of patients with CVA prior to and following therapy with inhaled corticosteroid and long-acting β2-agonist (ICS/LABA), were detected. The levels of IL-5 and percentage of eosinophils (EOS) in the induced sputum from patients with CVA were significantly higher than those in the control group of healthy individuals. The levels of MMP9 in the induced sputum from patients with CVA were also significantly higher than those in the control group. Following treatment with ICS/LABA for 6–9 months, the levels of MMP9 and IL-5, as well as the percentage of EOS, in the induced sputum from patients with CVA had significantly decreased. Thus, MMP9 may be an important biomarker in the airway inflammation of CVA.

## Introduction

Cough variant asthma (CVA) is a specific form of asthma that presents solely with cough. A previous study has revealed that ~30% of CVA may progress to classic asthma (1). CVA has many pathophysiological features that are similar to those of classic asthma, including airway hyper-responsiveness, atopy and chronic airway inflammation (2). Airway remodeling is an important feature of asthma and previous studies have confirmed its existence in patients with CVA (3,4). However, it remains to be determined whether there is a difference between classic asthma and CVA.

The matrix metalloproteinase (MMP) family is a protein family of zinc-dependent endopeptidases (5). MMPs are mainly involved in the cleavage of the extracellular matrix (ECM). They also play critical roles in a range of biological and pathological processes, including fibrosis, inflammation and the healing of wounds (6). A number of previous studies have revealed the critical role of MMPs in chronic obstructive pulmonary disease (COPD), interstitial lung disease (ILD), lung cancer and acute lung injury (7–10). Furthermore, several studies have demonstrated the important role of MMP9 in classic asthma (11–13). Patients with classic asthma have elevated levels of MMP9 in their serum, sputum and bronchoalveolar lavage fluid (BALF). MMP9 immunoreactivity has been demonstrated to be associated with the severity of classic asthma and MMP9-deficient animals exhibit reduced airway inflammation (5). However, one study reported heightened inflammation in MMP9-deficient mice, which suggests a protective role of MMP9 in classic asthma (14). Thus, it remains unknown whether MMP9 participates in the airway inflammation of CVA and whether this role is protective or sensitizing.

Thus, in the present study, the levels of MMP9 in the induced sputum of patients with CVA were detected. The effect of treatment with a combination of inhaled corticosteroid and long-acting β2-agonist (ICS/LABA) on MMP9 levels was also observed.

## Materials and methods

### Patients

Twenty-four patients with a clinical diagnosis of CVA were recruited from the Department of Respiratory Medicine of Jining First People’s Hospital (Jining, China). Thirty-one healthy individuals were simultaneously recruited as the control group. CVA was diagnosed according to recommendations in the Chinese national guidelines on the diagnosis and management of cough (15). None of the patients with CVA had previously received inhaled or oral steroids. Pregnant women, smokers and individuals who had had upper respiratory tract infections during the preceding two weeks were excluded. The individuals in the control group had no past history of asthma, atopic diseases or other respiratory diseases. All the patients with CVA received a therapy of ICS/LABA (salmeterol + fluticasone, 50 μg/250 μg bid; or fomoterol + budesonide, 4.5 μg/160 μg bid) for at least six months. Ethical approval was provided by the medical ethics committee of the Jining First People’s Hospital. Informed consent was obtained either from the patients or the patients’ families.

### Pulmonary function and bronchial provocation tests

All patients with CVA were diagnosed via the pulmonary function test (PFT) and bronchial provocation test (BPT). In the present study, all patients and control subjects performed a PFT. The PFT was carried out with a clinical spirometer (model MS-10S; Jaeger, Magdeburg, Germany) using a previously described method (11). Forced vital capacity (FVC) and forced expiratory volume (FEV1) measurements were taken and the FEV1/FVC ratio was calculated. The FEV1 percentage of predicted was calculated as the FEV1% predicted.

The BPT was performed using an aerodynamic particle-sized aerosol provocation system (model MS-10S; Jaeger). The provocation dose that caused a 20% reduction in FEV1 (PD20-FEV1) was measured through the inhalation of different concentrations of histamine. The baseline FEV1 was first measured using a spirometer. Patients inhaled a histamine aerosol from a nebulizer with tidal breathing whilst wearing a nose clip for 2 min. The total inhalations at each histamine concentration were administered and the FEV1 was measured three times following each period of inhalation. The BPT test was ceased if there was a reduction in the baseline FEV1 of 20% compared with that of the control inhalation solution. The subject would then be considered as airway hyper-responsive. Subjects received two puffs (200 μg) of salbutamol from a metered dose inhaler following the BPT.

### Induced sputum collection and counts

Induced sputum was collected with an aerosol of hypertonic saline solution according to a previous method (11). Subjects inhaled hypertonic saline (4%), which was delivered by an ultrasonic nebulizer (model 402AI, Yuyue, Jiangsu, China). Subjects were encouraged to cough and sputum was collected in clean polypropylene cups. The sputum specimen was examined within 2 h and prepared as previously described (11). A 1:10 dilution of dithiothreitol (DTT) was added in a volume equal to four times the weight of the selected sputum specimen. Samples were placed into a shaking water bath at 37°C for 15 min and subsequently further diluted with phosphate-buffered saline (PBS) to a volume equal to that of the sputum plus DTT. The suspension was filtered through a gauze to remove mucus and centrifuged at 120 × g for 8 min. The supernatant was aspirated and frozen at −80°C for subsequent analysis. Cell suspensions were adjusted to 1×10^5^/l and used for cytocentrifuge preparations. The slides were stained with a Giemsa stain and cell counts were performed under an optical microscope (model TS100, Nikon, Tokyo, Japan). The percentage of eosinophils (EOS) in each slide was calculated.

### Enzyme-linked immunosorbent assay (ELISA)

The MMP9 and interleukin (IL)-5 levels in the supernatant of the induced sputum were detected by commercial ELISA kits (R&D Systems, Minneapolis, MN, USA; Peprotech, Inc., Rocky Hill, NJ, USA, respectively) according to the manufacturers’ instructions. The detection limits were 0.5 ng/ml and 2 pg/ml, respectively.

### Statistical analysis

Data are expressed as means ± standard deviations and variables were assessed by the Kolmogorov-Smirnov test. The Mann-Whitney U or Kruskal-Wallis one-way analysis of variance tests were used for comparison in groups without a normal distribution. The Student’s t-test or analysis of variance (ANOVA) were used for comparison in groups with a normal distribution. P<0.05 was considered to indicate a statistically significant difference.

## Results

### Clinical data

The clinical characteristics of all subjects are shown in Table I. There were no significant differences in the age and gender of the two groups. The PFT results (including FEV1/FVC and FEV1% predicted values) in the two groups were similar. The percentage of EOS in the peripheral blood samples was also similar in the control and CVA groups. However, the total levels of immunoglobulin E (IgE) in the induced sputum of patients with CVA were significantly higher than those in the control group (565.2±46.5 vs. 133.5±15.4 IU/ml, respectively; P<0.01).

### IL-5 levels and EOS percentage in the induced sputum

**Figure 1:**
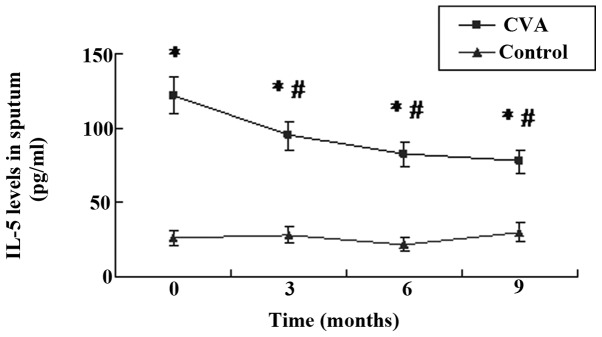
Levels of interleukin (IL)-5 in the induced sputum over time. Induced sputum was collected from the two groups and the level of IL-5 in the supernatant was detected by enzyme-linked immunosorbent assay (ELISA). ^*^P<0.05 as compared with the control group; ^#^P<0.05 as compared with the baseline; CVA, cough variant asthma.

**Figure 2:**
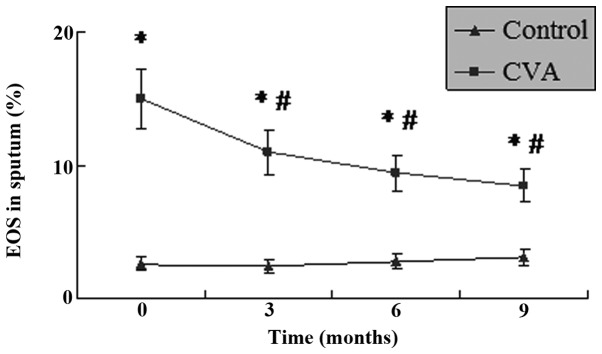
Percentage of eosinophils (EOS) in the induced sputum over time. Induced sputum was collected from the two groups. Cell suspensions were applied to slides and then stained with a Giemsa stain. The percentage of EOS in each slide was calculated under an optical microscope. ^*^P<0.05 compared with the control group; ^#^P<0.05 compared with the baseline; CVA, cough variant asthma.

As shown in Figs. 1 and 2, the levels of IL-5 and percentage of EOS in the induced sputum of patients with CVA were significantly higher than those in the controls (P<0.05). These results indicate that EOS-induced airway inflammation in CVA patients was similar to that in classic asthma. Following treatment with ICS/LABA for 3 months, the levels of IL-5 in the induced sputum of patients with CVA decreased significantly (P<0.05). The strongest inhibitory effect on IL-5 was exhibited following nine months of treatment. The EOS percentage followed a similar trend to the IL-5 level, and was significantly decreased following treatment with ICS/LABA (P<0.05). Although the IL-5 levels and EOS percentage decreased during the treatment, they remained significantly higher than those in the control group (P<0.05).

### MMP9 levels in the induced sputum

**Figure 3:**
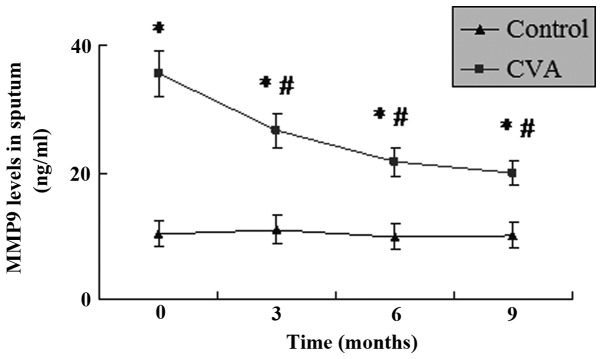
Levels of matrix metalloproteinase 9 (MMP9) in the induced sputum over time. Induced sputum was collected from the two groups and the level of MMP9 in the supernatant was detected with enzyme-linked immunosorbent assay (ELISA). ^*^P<0.05 as compared with the control group; ^#^P<0.05 as compared with baseline; CVA, cough variant asthma.

As shown in Fig. 3, the levels of MMP9 in the induced sputum of patients with CVA were significantly higher than those in the control group (P<0.05). Following treatment with ICS/LABA for different time periods, the level of MMP9 in the induced sputum of the patients with CVA decreased significantly. Although the level of MMP9 in the CVA group had decreased following treatment for 3 months, the strongest suppressing effect was revealed following 9–12 months. This range is due to the fact that the MMP9 levels of all the patients were collected at 9 months. However, several patients were lost at 12 months, therefore, the data at 9 months was shown but not at 12 months. However, the level of MMP9 remained higher than that in the control group for the duration of the study (P<0.05).

## Discussion

The present study revealed the presence of elevated levels of MMP9 in the induced sputum of patients with CVA and that treatment with ICS/LABA was able to significantly suppress airway inflammation and the levels of MMP9. The results indicate that MMP9 participates in the airway inflammation of CVA.

As CVA is a specific form of asthma, chronic airway inflammation is one of its most important features (2–4). Previous studies have revealed that numerous types of inflammatory factors or cytokines play a critical role in classic asthma and CVA, including IL-5, IL-4 and eosinophilic cationic protein (16,17). The present study demonstrated that the levels of IL-5 and the percentage of EOS increased in the induced sputum of patients with CVA. Treatment with ICS/LABA successfully inhibited the airway inflammation of CVA. These results confirmed the critical role of ICS/LABA in controlling the airway inflammation associated with CVA.

MMPs are mainly involved in ECM cleavage. They also play critical roles in a range of biological and pathological processes, including fibrosis, inflammation and the healing of wounds (6–10). A number of studies have demonstrated the presence of increased levels of MMP9 in the serum and sputum of patients with classic asthma (11–13). In addition, these increased levels have been shown to be associated with the FEV1 following allergen challenge. These results demonstrate the important role of MMP9 in the airway inflammation of classic asthma. The current study revealed elevated levels of MMP9 in the induced sputum of patients with CVA. Treatment with ICS/LABA was able to successfully reduce the percentage of EOS and also the levels of MMP9 and IL-5 in the sputum. This indicated that MMP9 may also participate in the chronic airway inflammation of CVA.

However, the precise role of MMP9 in chronic airway inflammation of CVA remains unclear. Certain studies have indicated that the upregulation of MMP9 may be associated with eosinophilia (18). The upregulation of MMP9 expression by EOS has also been demonstrated in nasal polyposis (19) and this may reveal an association between MMP9 and EOS. However, another study has demonstrated that MMP9 is not associated with the numbers of EOS or neutrophils (12). Therefore, further studies are required to explore the role of MMP9 in EOS and the airway inflammation of CVA.

## Figures and Tables

**Table I:** Clinical characteristics in the two groups.

Characteristic	Control group	CVA group	P-value
Number	31	24	
Gender (m/f)	18/13	14/10	>0.05
Age (years)	29.3±4.6	31.5±5.2	>0.05
FEV1/FVC	89.5±7.1	82.8±6.5	>0.05
FEV1% predicted	101.6±15.4	94.7±12.2	>0.05
Eosinophil (%)	3.3±0.4	4.1±0.5	>0.05
Total IgE (IU/ml)	133.5±15.4	565.2±46.5	<0.01

FEV1, forced expiratory volume; FVC, forced vital capacity; CVA, cough variant asthma; IgE, immunoglobulin E.
